# Evaluation of the Cerebral State Index in Cats under Isoflurane Anaesthesia: Dose-Effect Relationship and Prediction of Clinical Signs

**DOI:** 10.1155/2014/481460

**Published:** 2014-01-30

**Authors:** Joana R. Sousa, Lénio Ribeiro, Aura Silva, David A. Ferreira

**Affiliations:** ^1^Centro de Investigação em Ciências Veterinárias, Faculdade de Medicina Veterinária, Universidade Lusófona de Humanidades e Tecnologia, Campo Grande 376, 1749-024 Lisboa, Portugal; ^2^Hospital Veterinário do Porto, Travessa Silva Porto 174, 4250-475 Porto, Portugal; ^3^REQUIMTE, Laboratório de Toxicologia, Faculdade de Farmácia da Universidade do Porto, Rua de Jorge Viterbo Ferreira No. 228, 4050-313 Porto, Portugal

## Abstract

The performance of the cerebral state index (CSI) in reflecting different levels of isoflurane anaesthesia was evaluated in ten cats subjected to four end-tidal isoflurane concentrations (EtIso), each maintained for 15 minutes (0.8%, 1.2%, 1.6%, or 2.0% EtIso). The CSI, hemodynamic data, ocular reflexes, and eye position were recorded for each EtIso concentration. Pharmacodynamic analysis of CSI with EtIso was performed, as well as prediction probability analysis with a clinical scale based on the eye reflexes. The CSI values showed great variability. Between all parameters, burst suppression ratio showed the better fitting with the sigmoidal concentration-effect model (*R* ^2^ = 0.93) followed by CSI (*R* ^2^ = 0.82) and electromyographic activity (*R* ^2^ = 0.79). EtIso was the variable with better prediction of the clinical scale of anaesthesia (prediction probability value of 0.94). Although the CSI values decrease with increasing isoflurane concentrations, the huge variability in CSI values may be a strong limitation for its use in cats and it seems to be no better than EtIso as a predictor of clinical signs.

## 1. Introduction

Anesthetic depth monitoring is undergoing important innovations, especially with the recent introduction of new technological solutions, such as monitors that process the electroencephalographic (EEG) activity. In human anesthesia, such monitors are already part of the anesthetic monitoring equipment and are routinely used to assess depth of anesthesia. In animals, research has shown some potential of the EEG and its processed parameters to these applications [1–3]. In cats, there are only a few studies which evaluate the BIS for depth of anaesthesia assessment, by correlating its values with the anaesthetic concentration. In those studies, BIS values were inversely and linearly related to end-tidal sevoflurane [4] and isoflurane concentrations but were consistently low suggesting that clinical BIS endpoints used to titrate anesthetic agents in humans may not be applicable to cats [5]. This possibility was supported in a previous study, which suggests that clinical BIS endpoints used in humans may not be used in cats, due to the fact that BIS in this specie varied between 5 to 32 with 1.5 to 0.8 MAC isoflurane [5]. These limitations of the BIS monitor may be associated with the fact that its algorithm was developed based on EEG and clinical endpoints recorded in human patients and, consequently, may not be adapted to cats' EEG characteristics. Another limitation of the BIS monitor for veterinary use is its cost as well as the electrodes cost. A lower cost monitor such as the cerebral state monitor (CSM) could represent a more accessible alternative for veterinary use [6]. The CSM calculates cerebral state index (CSI), which, similarly to the BIS, is a dimensionless number that varies between 0 and 100. The manufacturer suggests that an index in the range from 40 to 60 corresponds to a surgical anesthetic level in humans [7]. Another potential advantage of this monitor is the fact that it was not developed based in data recorded in humans, and thus may apply better to cat EEG characteristics than the BIS.

The CSM monitor records the EEG waveform at a frequency of 2000 Hz and filters the signal between 6 and 42 Hz [7] It calculates the CSI based on frequency analysis of the EEG signal by characterizing the EEG energy into specific frequency bands. These are used to define two energy ratios: alpha (*α*) and beta (*β*). There is a shift exhibited in the energy content between *α* and *β* with deepening of anesthesia. The relationship between these shifts is also analysed by the CSM as a separate parameter (*β*-*α*). The monitor also evaluates the amount of burst suppression (periods of electroencephalographic isoelectricity alternated with high frequency and high amplitude bursts which are typical of very deep anesthesia) during each thirty second period of the EEG. The four parameters are then incorporated into a fuzzy-logic classifier system that calculates the CSI [7].

Isoflurane MAC value has been reported to be 1.61% in cats [8, 9] and its effects on the EEG of cats are well documented. In general, it causes a reduction in the EEG frequency (30 Hz in the waking state) to 12–16 Hz with increasing concentrations and at higher levels it causes burst suppression patterns. Cat anesthesia is often accomplished using a balanced technique, and this is most commonly achieved by combining inhalant anesthetics for immobility, muscle relaxation, and hypnosis with opioids for analgesia. However, it is important in a first phase to understand the effects of a single drug on the EEG and its derived parameters, before studying the effects of different drug combinations.

For the assessment of the performance of a monitor of anesthetic depth it is advised to analyze its correlation with the brain effect of the drug for its target concentration (or effect site concentration) as well as its correlation with the clinical status of the patient [10].

The objective of this study is to test the capacity of the CSI to reflect different concentrations of isoflurane in cats, by analyzing its correlation with the clinical signs used to assess the depth of anesthesia and the dose-effect relationship with isoflurane concentration.

## 2. Materials and Methods

### 2.1. Animals

This study involved 10 adult domestic cats (nine males and one female) from an animal rescue association, admitted to the Oporto Veterinary Hospital for routine surgery (orchiectomy and ovariohysterectomy) under a pet adopting program. The current study was authorized by the Portuguese authority for animal research (Direção Geral de Veterinária).

The animals were housed in individual cages in the animal ward of the veterinary hospital where they were monitored throughout the pre and postoperative period. In the preoperative period a complete physical exam was carried out in all of the cats, including preanesthetic haematology and biochemical blood analysis.

Exclusion criteria for this study included the administration of drugs with short or long term effects on the central nervous system (48 hours prior to the study), obvious mental status alterations, evidences of impaired hepatic or renal function, malnutrition, or other pathology that could compromise the patient's anesthetic safety. Female cats in heat or pregnant and male cats with abnormal external reproductive features, such as the absence of one or both of the testicles in the scrotum, were also excluded from analysis.

### 2.2. Hemodynamic and Respiratory Monitoring

The cephalic vein was catheterized with a 22 G catheter (0.9 × 25 mm, Introcan-W, Braun, Melsungen, Germany). An infusion pump (Infusomat FM, Braun, Melsungen, Germany) was used to administer 0.9% sodium chloride at rate of 10 mL/kg/h throughout the perioperative period.

A multiparameter anesthesia monitor Datex S/5 (Datex-Ohmeda, Helsinki, Finland) was used to monitor electrocardiogram, heart rate (HR), oxygen saturation of haemoglobin (SpO_2_), rectal temperature, end-tidal carbon-dioxide (EtCO_2_), inspired tidal fraction of oxygen, respiratory rate (RR), breathing pattern, and EtIso (%). To measure EtCO_2_ and EtIso, a sampling tube attached to the proximal end of the tracheal tube was connected to a Datex-Ohmeda Spirometry Module which determined the gas concentrations in the expired air.

Body temperature was kept constant with a homeothermic blanket (N-HB101-S-402, Panlab, Cornellà, Barcelona, Spain). The electrocardiogram electrodes were applied according to the specifications of the Academy of Veterinary Cardiology.

Noninvasive arterial pressures (systolic, diastolic, and mean arterial pressure—SAP, DAP, and MAP) were measured using the monitor petMAP (petMAP, Ramsey Medical Inc., Tampa, USA) with cuffs of appropriate size for each cat, following the recommendation of a cuff width equal to 40% of the circumference of the member to apply. The mean of four consecutive measurements (one measurement every 30 seconds) at each isoflurane concentration was used for analysis.

### 2.3. Brain Monitoring

Clamp electrodes as used in previous dog studies [2, 11–13]. Before placing the electrodes, the skin was degreased with 70% alcohol. Conductive gel (KY, Johnson and Johnson, Sezanne, France) was then applied in the recording area to provide better electrical conductivity and reducing the impedance between the electrodes and the skin. The electrodes were placed as described by Bollen and Saxtorph [14] for dogs along the median line of the skull. From rostral to caudal, the clamp corresponding to the negative electrode (black) was applied at the midline of the parietal bone, the clamp of the reference electrode (green) was located about 1 to 1.5 cm caudal to the previous electrode, and the clamp of the positive electrode (white) was applied at the intersection of the line that connects approximately one-third of the zygomatic process of frontal bone with external frontal crest.

The CSM used was the same as that employed in the previous studies [2, 12, 13]. It displays the EEG signal continuously and derives four parameters from the collected signal: the CSI, burst suppression (BS), electromyographic activity (EMG), and signal quality index (SQI). Further details on how these parameters are derived can be found in previous works [13]. CSI is based on a combination of four subparameters of the electroencephalographic signal: beta-ratio, alpha ratio, beta-alpha ratio, and BS. The BS is defined as the percentage of time in a 30-second window where the amplitude of the electroencephalographic signal was <3.5 *μ*V. These EEG periods characterize the deepest levels of hypnosis. Because high levels of facial muscular or EMG activity may interfere with the CSI under certain circumstances, the monitor incorporates an EMG filter that removes most of the potential interfering EMG activity. The EMG bar shows the energy of the EMG level in the 75–85 Hz frequency band (0–100 logarithmic scale).

### 2.4. Anesthetic Protocol

Cats were placed in a transparent induction chamber filled with a mixture of 5% isoflurane (IsoFlo, Esteve Pharma Ltd., Carnaxide, Portugal) in 100% oxygen for anaesthesia induction. When muscle relaxation was obvious, the animal was removed from the chamber and, after applying a spray of 10% lidocaine (Xylocaine 10%, AstraZeneca, London, England) directly on the arytenoids, a cuffed endotracheal tube with 2.5–3 mm diameter was inserted in the animal's trachea. After intubation, the cuff was inflated and adjusted to the inner diameter of the trachea. The endotracheal tube was then connected to the isoflurane vaporizer through a pediatric anaesthesia circuit (modified T-Ayres), delivering oxygen and 1% isoflurane in fresh oxygen at 1.5 L min^−1^. Cats were placed in right lateral recumbency, and the sensors for electroencephalographic, hemodynamic, and ventilatory monitoring connected.

Following the instrumentation phase, the study was started: animals were anaesthetized with four different EtIso: 0.8%, 1.2%, 1.6%, or 2.0% in a random order for each animal. After reaching the preselected EtIso, each cat was maintained at this EtIso concentration for 15 minutes to allow equilibrium between the blood and the effect compartment, after which the data was collected every 30 seconds for 2 minutes. The data collected included the parameters derived by the CSM, hemodynamic, and respiratory data, and ocular observations by a researcher blinded to the EtIso concentration. The cats were allowed to breathe spontaneously during the entire study.

The palpebral and corneal reflexes of the right eye were tested three times with a swab damped with sodium chloride 0.9%. The reflexes were considered negative if there were no response to these three stimulations. The palpebral reflex was tested gently at the lateral canthus of the eye, and the corneal reflex was elicited by a smooth pressure on the cornea. The eyeball position was observed. The palpebral reflex, defined by the partial or complete closure of the eyelids, is a commonly useful tool to help monitoring the depth of anesthesia. Its occurrence means that the patient is under a light plan of anaesthesia. If palpebral reflex is absent or depressed, the patient could be in a medium to a deep level of anesthesia [15]. The corneal reflex may also be used to help determining the depth of anesthesia. A corneal reflex is the eyelid response obtained to a careful and gentle touch in the cornea and should not cause any corneal damage. This reflex causes some controversy regarding its usefulness in accessing the depth of anesthesia. Some veterinary anaesthesiologists refer that corneal reflex is not a sign of deep anesthesia [15], while others report that this reflex should always be present, unless the patient is too deeply anaesthetised or dead.

During the entire study period, no stimulus was applied except those required for testing eye reflexes. The study was discontinued if one of the following situations occurred: apnoea (absence of inspiration for a period longer than 15 seconds) and hypotension (MAP lower than 60 mmHg during two consecutive measurements). If the animal exhibited clear signs of awakening (swallowing reflexes or increased mandibular tonus), the EtIso was increased, and the study was then continued with a different random EtIso.

At the end of the last EtIso studied, an intravenous 2.5 *μ*g/kg fentanyl followed by a constant rate infusion of 2.5 *μ*g kg^−1^ h^−1^ was slowly administered, and the isoflurane concentration was clinically adjusted. The patient was placed in dorsal recumbency, and the animals submitted to ovariohysterectomy or orchiectomy procedures.

### 2.5. Pharmacodynamic Analysis

To study the relationship between end-tidal isoflurane concentrations (EtIso) and the corresponding CSI, BS, and EMG values, the relation between EtIso and the three parameters was modeled using a sigmoidal *E*_max⁡_ model [16]:

(1) $$\begin{array}{c} E=E_{0}-E_{\max}\frac{c\gamma }{c\gamma +\text{E}{\text{C}}_{50}^{\gamma }}, \end{array}$$

where *E* is the pharmacodynamic effect (i.e., the CSI and clinical parameters), EC_50_ is the drug concentration that produces half-maximum effect, *E*_0_ is the effect at zero concentration, *E*_max⁡_ is the maximum effect, *c* is the drug plasma concentration effect, and the Hill exponent *γ* is a measure of curve steepness.

The pharmacodynamic parameters were estimated by population analysis using NONMEM (Globomax LLC, Hanover, MD). The goodness-of-fit for each index was compared using the values obtained for the coefficient of determination (*R* ^2^).

### 2.6. Statistical Analysis

The capacity of the studied parameters (CSI, EMG, BS, and EtIso) to detect the different anesthetic states, as reflected in the numerical scale of anesthesia was evaluated using prediction probability (Pk) statistics by correlating the parameter values with the numerical scale. Pk was calculated using a custom spreadsheet macro, PKMACRO [17]. A Pk of 1 means that the parameter always decreases as the subject reaches deeper anesthetic states. Alternatively, a Pk value of 0.5 or less would mean that the indicator is useless for predicting the depth of anesthesia. Pk was calculated using pooled data from all animals. The Pk value and the Jackknife SE is shown in Results. A delay of 50 seconds in the analysis of data from CSI data was considered [18].

Results are shown as mean ± standard deviation.

## 3. Results

Ten healthy adult cats (9 males and 1 female) weighing 3.25 ± 1.15 kg were analyzed. Hematologic or biochemical parameters were within the physiologic range in all animals.

During the fifteen minutes of stabilization of each isoflurane step, awakening occurred in all of the animals at 0.8% EtIso, and in six animals 1.2% EtIso. Because all of the cats awoke at 0.8% EtIso, it was not possible to analyze the data during the two minutes after the stabilization at this level and only the last values obtained in the 0.8% EtIso cats before awakening were used for analysis to represent the lowest concentration achieved in the study.

At 1.2% EtIso, only the data from the four cats that have not awakened were used for analysis.

At 1.6% EtIso, when compared with 0.8% EtIso, there was a decrease of 43% in the CSI, accompanied by a decrease of 88% in the EMG (Table 1), and a slight hemodynamic depression: −23% in MAP and −39% in HR (Table 1).

At 2.0% EtIso, hypotension occurred in three animals which were excluded from the analysis. Thus, only seven animals were considered for analysis in this step. There was a higher decrease in the CSI (−67%) and in the EMG (−99%), when compared to 0.8% EtIso. BS also increased in this step (+326%) (Table 1). The hemodynamic variables and RR showed a similar response as observed at 1.6% EtIso (Table 1).

**Figure 1:**
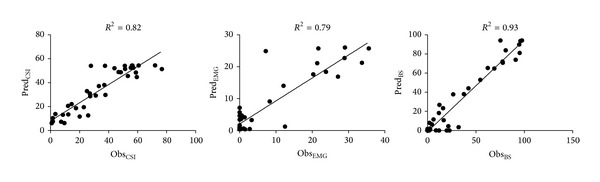
Representation of the observed effect (horizontal axis) versus the predicted effect (vertical axis) for the cerebral state index (CSI), the electromyographic activity (EMG), and the burst suppression ratio (BS). The coefficients of determination *R* ^2^ between the predicted and observed effects are also shown. Dots represent pooled data from all animals (*n* = 10) and the black line represents the regression line for the effects observed *versus* effects predicted by the inhibitory sigmoid *E*_max⁡_ model.

Pharmacodynamic analysis revealed better fitting of the *E*_max⁡_ model for BS (Figure 1) with a coefficient of determination (*R* ^2^) of 0.93, followed by CSI (*R* ^2^ = 0.82) and EMG (*R* ^2^ = 0.79). The estimated parameters *E*_0_, *E*_max⁡_, EC_50_, and *γ* for each of the studied indexes are shown in Table 2.

Three levels of depth of anaesthesia were identified based on clinical signs and a numerical scale was attributed to each depth to allow further prediction probability analysis. The absence of palpebral reflex was always associated with the absence of corneal reflex (Table 3).

The EtIso was the variable which showed better prediction of the clinical scale of anaesthesia followed by the EMG and the CSI. The results from prediction probability analysis between the levels of depth of anaesthesia and the other physiological parameters are shown in Table 4.

## 4. Discussion

These results show some potential of CSI to be used for monitoring of anaesthetic depth in cats. Regarding the concentration-effect relationship, CSI was inferior to BS which showed better fitting of the *E*_max⁡_ model. Concerning the prediction of the clinical signs of anaesthesia, the end-tidal isoflurane showed better prediction of the clinical signs than the CSI.

There was a great individual variability in the CSI values at each level of isoflurane, reflected by high standard deviation values, which may also affect the performance of this monitor.

The correlation with end-tidal isoflurane seems to be a more adequate method for assessing electroencephalogram-derived indexes performance than their prediction of clinical signs. The use of end-tidal isoflurane has important advantages over the clinical scale of anesthesia: it is an objective measure and it provides a continuous range of concentration values, which is important, as the electroencephalogram is also continuous [19]. Accordingly, the use of end-tidal anaesthetic has been proved as good as the BIS monitor for awareness prevention and anaesthetic adjustment in a large-scale study in humans [20]. However, it is important to note that the EtIso may be easily influenced by other factors, such as the combination of different drugs. In dogs, the combination of morphine, lidocaine, ketamine reduced isoflurane MAC, and consequently BIS was shown to increase as MAC of isoflurane decreased in all dogs from one study [21].

The first isoflurane concentration used in our study was an EtIso of 0.8%. As expected, all animals showed signs of awakening at this level. Thus, this level was considered as the level of awakening, and thus all other levels represent different states of progressive depression with increasing end-tidal concentrations of isoflurane. The results obtained for the 0.8% EtIso regarding the electroencephalographic parameters indicate that the CSI values set for these levels of hypnosis in humans (above 60) cannot be directly extrapolated to the feline population.

The mean CSI value obtained at this level in the cats in our study was 51.6, and all cats aroused. It is also important to note the large variability in CSI values observed at 0.8% EtIso, with a standard deviation of 23.9. This may be related either to the CSI algorithm or to interferences in the signal recorded by the monitor. At superficial depths of anaesthesia, the EMG levels were high due to animal movement during awaking, compromising the quality of the EEG signal recorded and, consequently, the CSI value displayed by the monitor. However, the fact that a mean value of BS of 14.3% was observed at 0.8% EtIso reveals a possible difficulty in the CSI algorithms to track the cat EEG patterns. The appearance of BS is characteristic of deep anaesthesia and has been reported in cats under isoflurane anaesthesia at levels of around 2.7% EtIso [5]. In this study, BS was detected by the CSM at levels in which the animals awoke. This was probably an error of the CSI algorithm in the evaluation of the EEG amplitude, in which it misinterpreted the low amplitude and high frequency awake EEG, with the low amplitude of a BS pattern.

At 1.2% EtIso there were six cases of awakening which were excluded from the final analysis. All variables were evaluated based only on the values of the four cats that have not awakened. Although none of the variables studied showed statistically significant differences with 0.8% EtIso, the mean CSI value was lower (36.1) and, according to the guidelines for humans, would be below the threshold defined as optimum surgical anesthetic level. However, this classification does not reflect the true clinical depth as shown by the presence of eyelid reflex and movement.

At 1.6% EtIso, the CSI value was around 30, equivalent to a very deep anaesthesia in the human scale. The significant reduction of EMG at this MAC value may be related to the absence of cases of awakening and intentional bodily movements when compared to 0.8% EtIso.

At 2.0% EtIso, the CSI indicated very deep anesthetic levels (mean values below 20), with a high BS value and a general depression of the hemodynamic and respiratory components, indicating that an EtIso of 2% is an excessive isoflurane concentration to be used in cats. This cardiorespiratory effect can be attributed to the dose-dependent depressive effects of isoflurane [9].

The results from this study are in agreement with a previous study with the BIS monitor [5]. In that study, all animals showed signs of shallow anaesthesia at 0.5 MAC (around 0.9% EtIso), and had burst suppression patterns at 2.0 MAC. Both the CSI and the BIS scales vary between 100 (awake) and 0 (brain silence), with 40–60 meaning adequate depth of anaesthesia. It is thus possible to compare the results of both studies in terms of CSI and BIS values. In our study, the maximum achieved was 2.0% EtIso, but the monitor detected burst suppression at the levels of anaesthesia. In the study by Lamont and colleagues [5] at 0.8 MAC (1.4% EtIso), the mean BIS was 32 while in our study at 0.8% EtIso mean CSI was 36; at 1 MAC (1.8% EtIso), mean BIS value was 20, and mean CSI value was 29 at 1.6% EtIso in the present study; at 1.5 MAC (2.7% EtIso), mean BIS value was 5, and in our study at 2.0% EtIso, CSI was 17.

*E*_0_ is the effect at zero concentration. In fact, in this study, pharmacodynamic modelling use the CSI data provided for the lower concentration which was EtIso of 0.8%. This may lead to a lower *E*_0_ than it would be expected if the fully awake CSI was recorded. However, for the determination of the quality of the dose-effect relationship through *R* ^2^ comparison, the actual dose (0.8) is used.

The predominant clinical signs of depth of anaesthesia observed in this study in cats under isoflurane anesthesia were the presence of eyelid and corneal reflexes with central eyeball, or the absence of eyelid and corneal reflex and ventromedial eyeball. It is important to highlight that the loss of eyelid reflex was always associated with the loss of corneal reflex. Thus, monitoring of anesthesia in cats based on these reflexes provides limited information and should be interpreted with caution and corroborated by additional information from monitoring other physiological variables. In this study, these clinical signs were analyzed as a numerical scale of only three levels, which may be a limitation. Nevertheless, the evaluation of the clinical signs was always performed by the same anesthesiologist and the conditions were the same for all the parameters studied by prediction probability analysis turning it possible to compare the different parameters.

In previous studies using the same monitor in dogs anesthetized with propofol, it was concluded that the inconsistency of the values of CSI with the clinical observations of different stages of anesthetic depth may limit the use of CSI as a monitor of depth of anesthesia in that species [13].

To the date, none of the monitors used to access depth of anaesthesia in humans provide reliable information when used to access depth of anaesthesia in cats. For the development of adequate measures of anaesthetic depth based on the EEG of cats it is essential to analyze the raw electroencephalogram and develop specific algorithms for these animals.

## 5. Conclusions

Although the CSI values decrease with increasing isoflurane concentrations, these values do not correlate progressively with the clinical signs of depth of anaesthesia. The huge variability in CSI values is a strong limitation for the use of CSI in cats, seriously compromising its reliability. The EtIso provides us a very good correlation with the clinical signs of depth of anaesthesia and should be used as an important tool to access depth of anaesthesia in cats. When monitoring the clinical signs of depth of anaesthesia in cats, it is important to take in consideration that the loss of corneal reflex occurs simultaneous with the loss of eyelid reflex.

## Figures and Tables

**Table 1:** Electroencephalographic parameters derived by the CSM and other physiologic data recorded at each end-tidal isoflurane concentration (EtIso—%) studied. The number of animals (*N*) at each EtIso step is shown. Mean ± standard deviation is shown.

EtIso (%)	*N*	CSI	BS (%)	EMG (%)	SQI (%)	MAP (mmHg)	HR (bpm)	SpO_2_ (%)	EtCO_2_ (%)	RR (rpm)	*T* (°C)
0.8	10	51.6 ± 23.9	14.3 ± 23.8	25.6 ± 28.5	79.5 ± 15.8	88.6 ± 30.6	163.0 ± 28.8	98.3 ± 1.3	36.7 ± 6.5	32.3 ± 12.7	37.4 ± 0.4
1.2	4	36.1 ± 28.3	36.2 ± 42.0	16.6 ± 14.1	81.2 ± 12.8	66.3 ± 14.4	138.3 ± 24.8	97.0 ± 1.2	36.5 ± 6.6	20.0 ± 2.9	37.1 ± 0.3
1.6	10	29.4 ± 13.5	25.7 ± 25.9	3.0 ± 8.5	84.3 ± 20.4	68.0 ± 24.3	140.2 ± 31.3	98.2 ± 1.0	39.1 ± 5.8	19.6 ± 6.6	37.2 ± 0.3
2.0	7	17.0 ± 17.7	60.9 ± 32.8	0.2 ± 0.6	91.0 ± 11.6	65.4 ± 13.1	144.3 ± 24.8	98.1 ± 1.3	42.6 ± 6.1	19.1 ± 6.3	37.2 ± 0.6

EtIso: end-tidal isoflurane concentration; CSI: cerebral state index; BS: burst suppression (%); EMG: electromyographic activity (%); SQI: signal quality index (%); MAP: mean arterial pressure (mmHg); HR: heart rate (bpm); SpO_2_: oxygen saturation of haemoglobin (%); EtCO_2_: end-tidal carbon dioxide (%); RR: respiratory rate (rpm); *T*: temperature (°C).

**Table 2:** Results from the pharmacodynamic modeling for the parameters studied.

	*E*_max⁡_	EC_50_	*E*_0_	*γ*
CSI	6.11	1.53%	54.4	7.3
BS	9.4%	1.75%	0%	9.95
EMG	0.38%	1.04%	26.1%	8.61

Data are estimate ± standard deviation. CSI: cerebral state index; BS: burst suppression ratio; EMG: electromyographic activity; *E*_max⁡_: maximum effect; EC_50_: concentration at 50% maximum effect; *E*_0_: baseline effect; *γ*: hill exponent.

**Table 3:** Number of cats that achieved each anaesthetic plane for each end-tidal isoflurane (EtIso—%) concentration studied.

EtIso—%	Ocular reflexes	Attributed numerical scale	Number of cats that achieved the plane/total number of cats
0.8	PR+/CR+/EC	1	10/10
1.2	PR+/CR+/EC	1	7/10
	PR+/CR+/EV	2	1/10
	PR−/CR−/EV or EC	3	2/10
1.6	PR+/CR+/EC	1	1/10
	PR+/CR+/EV	2	1/10
	PR−/CR−/EV or EC	3	8/10
2.0	PR−/CR−/EV or EC	3	10/10

A numerical scale was attributed from 1 to 3 according to the anaesthetic depth based on the presence (+) or absence (−) of ocular reflexes and the position of the eye: PR: palpebral reflex; CR: corneal reflex; EC: eyeball centred in the eye; EV: eyeball rotated ventrally.

EtIso: end-tidal isoflurane concentration; numerical scale attributed according to the ocular reflexes observed.

**Table 4:** Results from prediction probability (Pk) analysis calculated between the clinical scale of anaesthesia (Table 3) and the different parameters studied, from pooled data of the 10 cats. The Pk value and the Jackknife standard deviation (SE) are shown.

Parameter	Pk (SE)
EtIso	0.94 (0.03)
CSI	0.79 (0.07)
EMG (%)	0.83 (0.08)
BS (%)	0.75 (0.08)

EtIso: end-tidal concentration of isoflurane; CSI: cerebral state index; EMG: electromyographic activity (%); BS: burst suppression (%).
